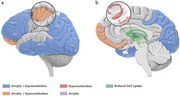# The role of neuroimaging in identifying prodromal Dementia with Lewy bodies: insights on anatomo‐functional brain changes and clinical implications

**DOI:** 10.1002/alz70856_102819

**Published:** 2025-12-24

**Authors:** Desirée Conti, Giulia Bechi Gabrielli, Massimiliano Panigutti, Giulia Zazzaro, Giuseppe Bruno, Gaspare Galati, Fabrizia D'Antonio

**Affiliations:** ^1^ Fondazione Santa Lucia IRCCS, Rome, Italy; ^2^ Sapienza University of Rome, Rome, Italy

## Abstract

**Background:**

Prodromal Dementia with Lewy bodies (pro‐DLB) has been recently defined; however, the neuroanatomical and functional correlates of this stage have not yet been univocally established. This study aimed to systematically review neuroimaging findings focused on pro‐DLB.

**Method:**

We performed a literature search using PubMed and Scopus and retrieved 8956 unique records employing MRI, PET and SPECT to examine pro‐DLB patients. After applying inclusion and exclusion criteria, 40 records were included in the systematic review: 15 studies assessed structural gray matter (GM) and white matter (WM) integrity, and 31 investigated functional metabolism, perfusion, and resting‐state connectivity.

**Result:**

In pro‐DLB, frontal lobe areas (including the prefrontal cortex) were characterized by decreased function, cortical atrophy, and WM damage. Volumetric reductions were found in the insula, which also showed heightened metabolism. A pattern of hypofunction and structural damage characterized the lateral and ventral temporal lobe, including the fusiform and lingual gyri; instead, the parahippocampal cortex and hippocampus exhibited greater function. Hypometabolism and hypoperfusion marked parietal and occipital regions, with localized atrophy in the medial occipital lobe (i.e., the visual system) and the posterior parietal cortex (encompassing the precuneus and inferior parietal lobule). Less consistent results were found regarding the cingulate island sign. Subcortically, atrophy and microstructural damage in the nucleus basalis of Meynert were reported, and dopamine transporter uptake was reduced in the basal ganglia. The main results are presented in Figure 1.

**Conclusion:**

Structural and functional damage was already present in the prodromal stage of DLB and was coherent with the possible clinical onset. Frontal and parieto‐occipital alterations may be associated with deficits in attention and executive functions and in visuo‐perceptual/visuo‐spatial abilities, respectively. Degeneration of cholinergic and dopaminergic transmission appeared substantial at this disease stage. The review highlighted the clinical usefulness of the neuroimaging biomarkers proposed for this population of patients. Thus, the review provided an updated and more precise depiction of the brain alterations that are specific to pro‐DLB and valuable to its differentiation from physiological aging and the predementia stage of other diseases, especially MCI due to AD.